# Contrast enhanced CMR in acute myocarditis: what is the optimal moment for imaging?

**DOI:** 10.1186/1532-429X-13-S1-O36

**Published:** 2011-02-02

**Authors:** Alexis Jacquier, Nicolas Amabile, Jean Yves Gaubert, Francesca Carta, Antonin Flavian, Boris Maurel, Guy Moulin

**Affiliations:** 1 CHU La Timone, CEMEREM, Marseille, France; 2 Marielanelongue, Paris, France; 3 CHU La Timone, Marseille, France

## Introduction

The pathological processes (ie alteration of myocardial perfusion) that occur during the acute phase of a myocarditis, are different from those of myocardial infarction. Myocardial enhancement kinetics might be different in acute myocarditis compared to myocardial infarct that may impact the optimal moment for imaging acute myocarditis.

## Purpose

To compare the kinetics of gadolinium in acute myocarditis and acute myocardial infarction and 2) to establish the best interval between contrast injection and delayed contrast enhanced (DCE) image acquisition for the diagnosis of acute myocarditis.

## Methods

Ninety-nine patients were prospectively included, 17 patients with acute myocarditis and 12 with acute myocardial infarction. All patient underwent a 1.5T CMR examination. Look-Locker sequences were acquired before and after administration of 0,2mol/kg Gd-DOTA repeatedly during 14min with the following parameters: TR/TE=26.7/1.27 ms, slice thickness= 8mm, α=30°, matrix=192 x 96, 26 cardiac triggered images were acquired following each non-selective inversion pulse with TI_0_=95ms, ΔTI=25ms. The apparent longitudinal relaxation rate (R’1) from left ventricular blood, enhanced and normal myocardium and were calculated. Delayed contrast enhanced (DCE) images were acquired at 5, 10 and 15min after contrast injection (TR/TE=650 / 1.56 ms, slice thickness=7mm, spacing=0, α=10° , and matrix =152x134. The inversion time=270-325ms. Signal noise ratio (SNR) and contrast noise ratio (CNR) were measured and a qualitative image analysis was performed on a four points scale.

## Results

**Figure 1:**
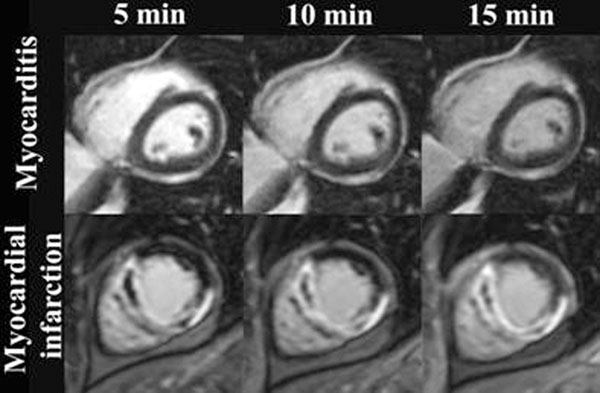
DCE images 5, 10 and 15min in one patient with myocarditis (upper row) and in one other patient with myocardial infarction (lower row).

A faster decline in R’1 value was measured within the area of myocardial enhancement in myocarditis compared with myocardial infarction. Myocarditis patients showed a significant decrease in CNR (from 92±58 at 5min vs 64±47 at 15min; P<0.0001) as a function of time (Figure 1). Whereas in myocardial infarction, there was no significant difference between time points for CNR (92±57 at 5min vs 100±50 at 15min; P=ns). Image quality in myocarditis was significantly better at 5 min (3.6±0.6) and decreased over time at 10 (2.7±0.5) and 15min (2.1±0.7; P=0.01) after contrast injection (figure 1).

## Conclusions

The gadolinium kinetics of acute myocarditis are different from those of acute myocardial infarction. In myocarditis, DCE images acquired 5min after contrast injection provide higher SNR, CNR and image quality compared with images collected at later times.

